# Exploring the Structural and Electronic Properties of Niobium Carbide Clusters: A Density Functional Theory Study

**DOI:** 10.3390/molecules29133238

**Published:** 2024-07-08

**Authors:** Hui-Fang Li, Huai-Qian Wang, Yu-Kun Zhang

**Affiliations:** 1 College of Engineering, Huaqiao University, Quanzhou 362021, China; 2 College of Information Science and Engineering, Huaqiao University, Xiamen 361021, China

**Keywords:** density functional theory, structure, stability, electronic property, ab initio molecular dynamics

## Abstract

This paper systematically investigates the structure, stability, and electronic properties of niobium carbide clusters, Nb_m_C_n_ (m = 5, 6; n = 1–7), using density functional theory. Nb_5_C_2_ and Nb_5_C_6_ possess higher dissociation energies and second-order difference energies, indicating that they have higher thermodynamic stability. Moreover, ab initio molecular dynamics (AIMD) simulations are used to demonstrate the thermal stability of these structures. The analysis of the density of states indicates that the molecular orbitals of Nb_m_C_n_ (m = 5, 6; n = 1–7) are primarily contributed by niobium atoms, with carbon atoms having a smaller contribution. The composition of the frontier molecular orbitals reveals that niobium atoms contribute approximately 73.1% to 99.8% to Nb_m_C_n_ clusters, while carbon atoms contribute about 0.2% to 26.9%.

## 1. Introduction

Previous studies have indicated that detailed experimental and theoretical investigations of transition metal clusters often reveal remarkable relationships between their physical properties and chemical reactivity [1,2,3,4]. Niobium clusters are among the most interesting transition metal clusters that have been extensively studied. They possess a long and rich history of experimental and theoretical investigations, attributed to several special properties, including the relative tendency to form clusters, the existence of a single naturally occurring isotope, high melting point, high temperature resistance, and superconductivity [5,6,7,8,9,10,11,12,13,14,15,16,17,18,19,20,21,22,23,24]. The relative propensity to form clusters is experimentally advantageous, and the presence of only one naturally occurring isotope simplifies the interpretation of niobium spectra by avoiding complications from overlapping features caused by different isotopomers. On the experimental side, the ionization potential (IP) [5], dissociation energy (DE) [6,7,8], reactivity [9,10,11], and electron affinity (EA) [12,13,14] have been measured on niobium clusters and their ions. The results have revealed dramatic size-dependent fluctuations of the electronic and chemical properties of niobium clusters. For example, both the reactivity rates of Nb_n_ (n ≤ 20) clusters with D_2_ and the ionization potential show that Nb_8_, Nb_10_, and Nb_16_ are relatively unreactive [15,16]. The strong size dependence of their chemical reactivity further makes them attractive for study, as certain species may possess the required balance of properties for nanotechnology applications. On the theoretical side, niobium has garnered significant attention, with a focus on studying the structural and electronic properties of a series of pure niobium or niobium-related clusters through the application of density functional theory (DFT) [19,20,21]. Many studies on small Nb clusters confirm that Nb_8_, Nb_10_, and Nb_16_ clusters have higher stability than other clusters. Pansini and co-workers studied the electronic structure and electrical properties of Al-doped niobium clusters [21]. By using all-electron density functional theory with Douglas–Kroll–Hess correction, the spin state, geometry, hardness, and mean static dipole polarizability of Nb_x_Al_y_ clusters were examined.

Among the studies, niobium carbides, Nb_m_C_n_, have been the topic of extensive theoretical and experimental research [25,26,27,28,29,30,31,32,33,34,35] due to their potential applications in numerous areas, including catalysis, electronic materials, and energy storage. Mass spectrometry [25,26,27,28], X-ray diffraction spectroscopy [29,30], photoelectron spectroscopy [30,31,32], and electron spin resonance spectroscopy [33], combined with DFT, have been applied to study niobium carbide clusters. The mass spectroscopic studies by Mafuné and co-workers used a double laser ablation technique, and ionization potentials (IPs) of a series of Nb_n_C_m_ (n = 3–10, m = 0–7) were measured and compared with the IPs estimated by density functional theory calculations [28]. Electron spin resonance spectroscopy was performed to study diatomic NbC by Hamrick and Weltner [33]. The elastic properties of niobium carbide NbC_y_ were studied by Gusev and co-workers [34]. Metha and co-workers used photoionization efficiency spectroscopy in combination with DFT to determine the ionization potential of Nb_3_C_n_ (n = 1–4) and Nb_4_C_n_ (n = 1–6), and Nb_3_C_2_ and Nb_4_C_4_ clusters exhibited the lowest IPs for the two series, respectively [27].

In this study, we investigate the geometry, stability, and electronic structures of Nb_m_C_n_ (m = 5, 6; n = 1–7) clusters using DFT calculations with the BPW91 functional. Firstly, a global search is performed to obtain the geometric structures of Nb_5_C_n_ and Nb_6_C_n_ (n = 1–7) clusters. Secondly, the relative stability of the optimized structure is determined by analyzing the average binding energy, dissociation energy, and second-order difference energy, and the stability reasons are explained. Finally, the density of states and compositions of the frontier molecular orbitals are discussed.

## 2. Results and Discussion

### 2.1. Geometry

**Figure 1:**
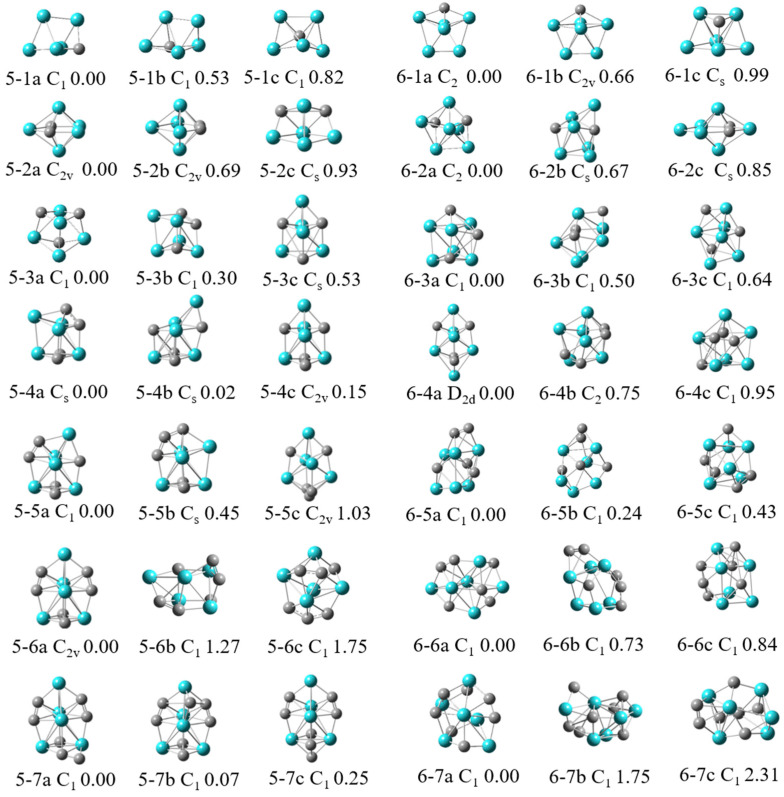
Geometric structures, point group symmetries, as well as relative energies of Nb_5_C_n_ and Nb_6_C_n_ (n = 1–7) clusters. The relative energies are in eV.

The structures of different isomers for Nb_5_C_n_ and Nb_6_C_n_ (n = 1–7) are obtained using the BPW91 functional with the 6-311G+(2d) basis set for C and SDD basis set for Nb. Figure 1 displays the lowest-energy geometries and some low-lying isomers of Nb_5_C_n_ and Nb_6_C_n_ (n = 1–7). According to the total energy from low to high, the isomers of Nb_5_C_n_ (n = 1–7) are designated by 5-na, 5-nb, and 5-nc, while the isomers of Nb_6_C_n_ (n = 1–7) are designated by 6-na, 6-nb, and 6-nc, where n is the number of C atoms. The Cartesian coordinates of the lowest-energy structures of Nb_5_C_n_ and Nb_6_C_n_ (n = 1–7) are summarized in Table S1 of the Supplementary Materials. The optimized results show that the most stable structures of Nb_5_C_n_ (n = 1–7) exhibit a doublet spin state, whereas those of Nb_6_C_n_ (n = 1–7) exhibit a singlet spin state. In the following sections, we discuss these results in detail.

#### 2.1.1. Nb_5_C_n_ (n = 1–7)

The lowest-energy structure of Nb_5_C is found to be a distorted prism with a doublet spin state, which can be viewed as the one obtained from replacing one Nb atom by a C atom in the Nb_6_ structure. The corresponding quartet states are higher in energy than the doublet state by 0.26 eV. Another two similar structures, 5-1b and 5-1c, are obtained, and their relative energies to the ground state are 0.53 and 0.82 eV, respectively.

Nb_5_C_2_ is an irregular pentagonal bipyramid with similar features as the structure of Nb_7_. The structure of Nb_5_C_2_ can be described as the one obtained from replacing two Nb atoms by C atoms sitting on the pentagonal ring. The point group symmetry is C_2v_ and the calculated equatorial bond lengths of the pentagonal ring are 2.04, 2.05, and 2.84 Å, whereas the polar-equatorial bond lengths are 2.19, 2.63, and 2.55 Å. The pole-to-pole bond length is 3.13 Å. Structure 5-2b is also a pentagonal bipyramid with C_2v_ point group symmetry. Comparing structures 5-2a and 5-2b, the two C atoms occupy opposite positions in the pentagonal ring in structure 5-2a and adjacent positions in structure 5-2b. Structure 5-2b lies 0.69 eV above structure 5-2a. Structure 5-2c possesses low C_s_ symmetry and is less stable than 5-2a by 0.93 eV.

A previous study predicted that Nb_5_C_3_ has a similar cubic structure as Nb_4_C_4_. The BPW91 calculation determined that Nb_5_C_3_ is a distorted cubic structure and has low C_1_ symmetry. Structure 5-3a consists of two quadrangles. The upper quadrangle contains two C atoms in opposite positions and the lower quadrangle has one C atom. Isomer 5-3b presents a similar geometry as structure 5-3a, with a slight distortion, but with two C atoms occupying adjacent positions. Isomers 5-3b and 5-3c are less stable than 5-3a by 0.30 and 0.53 eV, respectively.

The three low-lying isomers of Nb_5_C_4_ listed in this paper are all capped structures, and the main difference between the three isomers is the position of the four C atoms in the cluster. It should be pointed out that the energies of structures 4a and 4b are very close, and the energy of structure 5-4b is only 0.02 eV higher than that of 5-4a. In view of such a small relative energy and in order to ensure the accuracy of calculations, we conducted DFT computations with the Def2-DZVPP basis set instead of the SDD basis set. It was found that the energy difference between 5-4a and 5-4b was very small, but the energy level order was reversed. The energy of structure 5-4b was 0.01 eV lower than that of the 5-4a structure (see Table S2). Additionally, DFT calculations were also performed using the B3LYP functional. The B3LYP functional calculations showed that isomer 5-4b is the lowest energy structure, in which each of the two C atoms occupies the opposite position of the upper and lower quadrangle. In this case, we calculated the vertical ionization potentials (VIPs) of both isomers 5-4a and 5-4b. The results found that the calculated VIP of isomer 5-4b is 5.15 eV (B3LYP) or 5.13 eV (BPW91), and the calculated VIP of isomer 5-4a is 4.85 eV (B3LYP) or 4.90 eV (BPW91). No matter the B3LYP or BPW91 functional, the calculated VIP value for structure 5-4b is in better agreement with the experimental result (5.12 ± 0.12 eV) than for structure 5-4a. Therefore, isomer 5-4b should be the ground state structure. Isomer 5-4c is less stable than 5-4a by 0.15 eV.

From the point of view of growth, the lowest-energy structure of Nb_5_C_5_ can be viewed as the one obtained from capping of the C atom to the lowest-energy structure of Nb_5_C_4_ with a little local distortion. Another isomer, 5-5b, which possesses similar shape but a different C atom position with isomer 5-5a, is less stable than 5-5a by 0.45 eV. Structure 5-5c, which has relatively high C_2v_ point group symmetry and can be obtained by adding one C atom to the bottom of structure 5-4c, is 1.03 eV higher in energy than structure 5-5a.

The lowest-energy structure for Nb_5_C_6_ is found to be a ring, consisting of four C atoms and three Nb atoms capped with one C atom and one Nb atom on each side of the ring. The point group symmetry is C_2v_. Another two structures, 5-6b and 5-6c, with lower C_1_ symmetry, are less stable than structure 5-6a by 1.27 and 2.22 eV, respectively. For Nb_5_C_7_, when a C atom is added to the lowest-energy structure of 5-6a at different positions, isomers 5-7a, 5-7b, and 5-7c are formed. Isomer 5-7a is more stable than 5-7b and 5-7c by 0.07 and 0.25 eV, respectively. 

#### 2.1.2. Nb_6_C_n_ (n = 1–7)

For Nb_6_C, the lowest-energy structure is a pentagonal bipyramid with four Nb atoms and one C atom in a plane, and the other two Nb atoms are located on each side of the pentagonal ring. The energies of the other two structures, 6-1b and 6-1c, are higher than those of structure 6-1a by 0.66 eV and 0.99 eV, respectively. We found the lowest-energy structure of Nb_6_C_2_ is approximately C_2_ point group symmetry. Three Nb atoms and two C atoms make up a ring that is not in the plane, and one Nb atom and a dimer Nb_2_ occupy each side of the ring, respectively. Another structure, 6-2b, with C_s_ point group symmetry, is less stable than 6-2a by 0.67 eV. The lowest-energy structure of Nb_6_C_3_ can be obtained by capping of an atom to Nb_5_C_3_ with different positions of C atoms. Structures 6-3b and 6-3c are less stable than 6-3a by 0.50 and 0.64 eV, respectively. The lowest-energy structure 6-4a has relatively high D_2d_ point group symmetry. The structure can be viewed as the one obtained from capping of a Nb atom to the bottom of structure 5-4c of Nb_5_C_4_, with small structure distortion. Structures 6-4b and 6-4c are 0.75 and 0.95 eV higher in energy than structure 6-4a. As for Nb_6_C_5_, Nb_6_C_6_, and Nb_6_C_7_ clusters, three low-lying isomers of each system are listed in this paper; isomer 6-5a is the structure with the lowest energy, and the energies of the other two isomers are 0.24 and 0.43 eV higher than that of structure 6-5a, respectively. Isomer 6-6a is the lowest-energy structure for the Nb_6_C_6_ cluster, and isomers 6-6b and 6-6c are less stable than isomer 6-6a by 0.73 and 0.84 eV, respectively. Isomers 6-7b and 6-7c are less stable than the lowest-energy isomer 6-7a by 1.75 and 2.31 eV, respectively.

### 2.2. Comparison between Calculated and Experimental Vertical Ionization Potential

**Figure 2:**
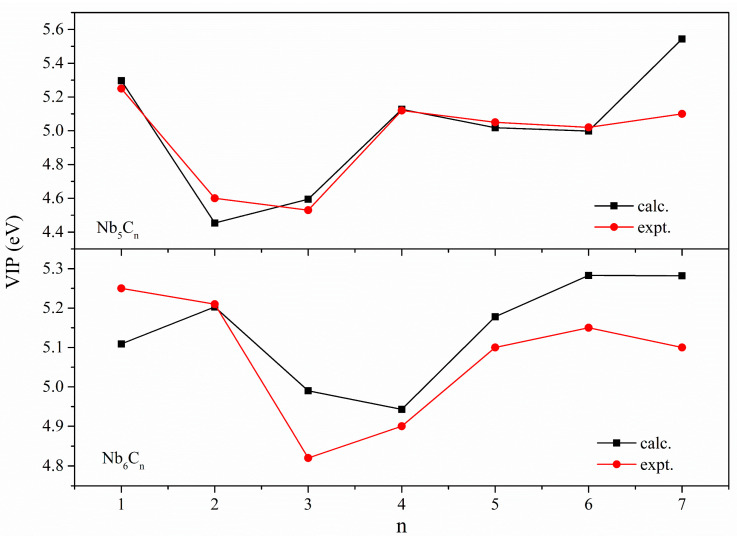
The experimental and calculated VIPs of Nb_5_C_n_ and Nb_6_C_n_ (n = 1–7) clusters.

The VIPs of the stable structures of Nb_5_C_n_ and Nb_6_C_n_ (n = 1–7), determined with BPW91 calculations, are shown in Table 1 and Figure 2, along with the experimental results [28]. The calculated VIP values are in excellent agreement with the experiments for Nb_5_C_n_ (n = 1–6); however, for Nb_5_C_7_, the calculated result likely overestimates this parameter, and the relative error is 8.6%. We tried to obtain more of the various structures and calculate the VIPs of different isomers, but the results were not satisfactory. For Nb_6_C_n_ (n = 1–7) clusters, the employed DFT method predicted VIPs with acceptable agreement with the experiments. The calculation results showed that the relative error of Nb_6_C_n_ (n = 1–7) clusters is within 3.5%. The good agreement between the calculated VIPs and the experimental values gives confidence in the assigned the ground state for the complexes considered in the present paper.

### 2.3. Relative Stability

To determine the structural stabilities of these Nb_5_C_n_ and Nb_6_C_n_ (n = 1–7) clusters, the average binding energy per atom (E_b_) can be calculated using the following formulas:

E_b_ (Nb_5_C_n_) = [5E(Nb) + nE(C) − E(Nb_5_C_n_)]/(5 + n) (1)

E_b_ (Nb_6_C_n_) = [6E(Nb) + nE(C) − E(Nb_6_C_n_)]/(6 + n) (2)

**Figure 3:**
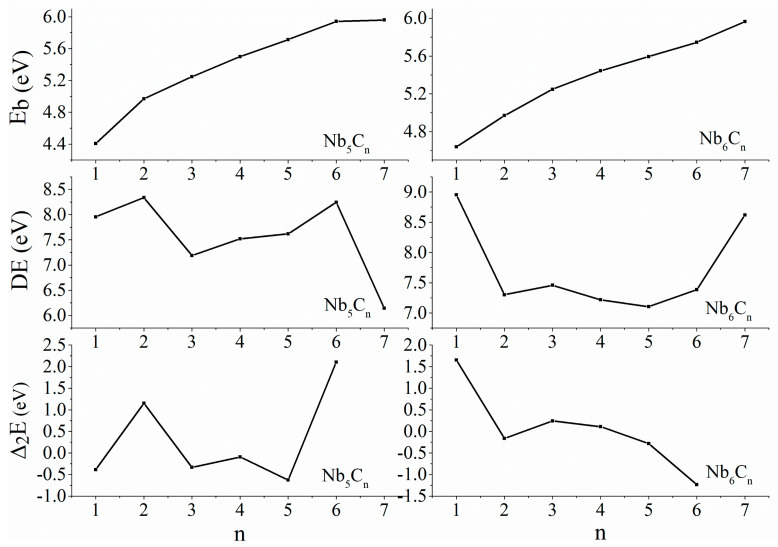
The average binding energy per atom (E_b_), dissociation energy (DE), and second-order difference energy ($\Delta_{2}E$) for Nb_5_C_n_ and Nb_6_C_n_ (n = 1–7) clusters.

Here, E(Nb), E(C), E(Nb_5_C_n_), and E(Nb_6_C_n_) represent the total energy of Nb, C, Nb_5_C_n_, and Nb_6_C_n_, respectively, where “n” denotes the number of C atoms. The average binding energy is a measurement of the thermodynamic stability of the clusters, and the results are listed in Table 2 and plotted in Figure 3 as a function of the number of C atoms. As shown in Figure 3, the E_b_ values of these Nb_5_C_n_ and Nb_6_C_n_ (n = 1–7) clusters are quite large, ranging from approximately 4 to 6 eV, and are close to each other. For Nb_5_C_n_ (n = 1–7), the E_b_ becomes a growing function of the number of C atoms and has two inflection points, corresponding to n = 2 and 6, indicating that Nb_5_C_2_ and Nb_5_C_6_ clusters are more stable than others. For the Nb_6_C_n_ cluster, the E_b_ shows a monotonic increasing trend with increasing cluster size. Starting from n = 3, the rate of increase in average binding energy slightly decelerates, indicating that Nb_6_C_3_ exhibits slightly higher but not significantly pronounced stability.

The stability of Nb_5_C_n_ and Nb_6_C_n_ (n = 1–7) clusters was further investigated by calculating the dissociation energy (DE), defined as follows:

DE (Nb_5_C_n_) = E (Nb_5_C_n−1_) + E(C) − E(Nb_5_C_n_) (3)

DE (Nb_6_C_n_) = E (Nb_6_C_n−1_) + E(C) − E(Nb_6_C_n_) (4)

The dissociation energies to remove a C atom from the clusters are illustrated in Table 2 and Figure 3. For the Nb_5_C_n_ (n = 1–7) cluster, the dissociation energies of Nb_5_C_2_ and Nb_5_C_6_ corresponding to dissociation paths $Nb_{5}C_{2}\to Nb_{5}C+C$ and $Nb_{5}C_{6}\to Nb_{5}C_{5}+C$ yield the local maxima of all calculated clusters, indicating that Nb_5_C_2_ and Nb_5_C_6_ clusters are more stable than their neighbors. The dissociation energy of Nb_5_C_3_ corresponding to the dissociation path $Nb_{5}C_{3}\to Nb_{5}C_{2}+C$ yields the local minima of all calculated clusters, indicating that Nb_5_C_3_ has relatively weak stability. For Nb_6_C_n_, the dissociation energy of Nb_6_C_3_ corresponding to the dissociation path $Nb_{6}C_{3}\to Nb_{6}C_{2}+C$ yields a small local maximum, indicating slightly higher stability compared to neighboring clusters. This result is consistent with the results of the average binding energy.

The second-order difference (Δ_2_E) refers to taking the difference again based on the first-order difference of energy. Δ_2_E, a more sensitive quantity, reflects the relative stability of the clusters. Positive peaks of Δ_2_E indicate greater stability of the cluster, while a smaller Δ_2_E suggests weaker stability of the cluster. The Δ_2_E values of Nb_5_C_n_ and Nb_6_C_n_ (n = 1–7) clusters were determined using the formulas:

Δ_2_E (Nb_5_C_n_) = E(Nb_5_C_n−1_) + E(Nb_5_C_n+1_) − 2E(Nb_5_C_n_) (5)

Δ_2_E (Nb_6_C_n_) = E(Nb_6_C_n−1_) + E(Nb_6_C_n+1_) − 2E(Nb_6_C_n_) (6)

where E(Nb_5/6_C_n−1_), E(Nb_5/6_C_n_), and E(Nb_5/6_C_n+1_) represent the total energy of Nb_5/6_C_n−1_, Nb_5/6_C_n_, and Nb_5/6_C_n+1_, respectively. The Δ_2_E is presented in Table 2 and plotted in Figure 3 as a function of the number of C atoms. The minima of Nb_5_C_n_ (n = 1–7) are found at Nb_5_C_3_ and Nb_5_C_5_, indicating that these clusters show obviously weak stability. The maxima of Nb_5_C_n_ are found at n = 2 and 6, indicating that Nb_5_C_2_ and Nb_5_C_6_ possess higher stability than their neighbors. The curve for DE shows a similar behavior in Figure 3, confirming the thermodynamic stability of these clusters. For Nb_6_C_n_ (n = 1–7), Δ_2_E initially decreases, then increases, and gradually decreases again from n = 3 to 6. The curve of Δ_2_E reaches a small local maximum at n = 3, showing a trend similar to the dissociation energy, implying that the stability of Nb_6_C_3_ clusters is slightly higher than that of neighboring clusters. 

Comparing the analysis results of average binding energy, dissociation energy, and second-order difference energy, it can be observed that Nb_5_C_2_ and Nb_5_C_6_ exhibit significantly higher stability. The stability of Nb_6_C_3_ is slightly higher among Nb_6_C_n_ clusters but not notably so. Next, we use ab initio molecular dynamics (AIMD) analysis to explain the reasons for the higher stability of Nb_5_C_2_ and Nb_5_C_6_. Furthermore, when n = 1 and n = 7, due to the limitations imposed by the cluster sizes computed in this study, Nb_5_C and Nb_5_C_7_ as well as Nb_6_C and Nb_6_C_7_ are positioned at the endpoints of the line. Their stability will also be further discussed in conjunction with AIMD analysis.

### 2.4. Stability Analysis via AIMD Simulations

**Figure 4:**
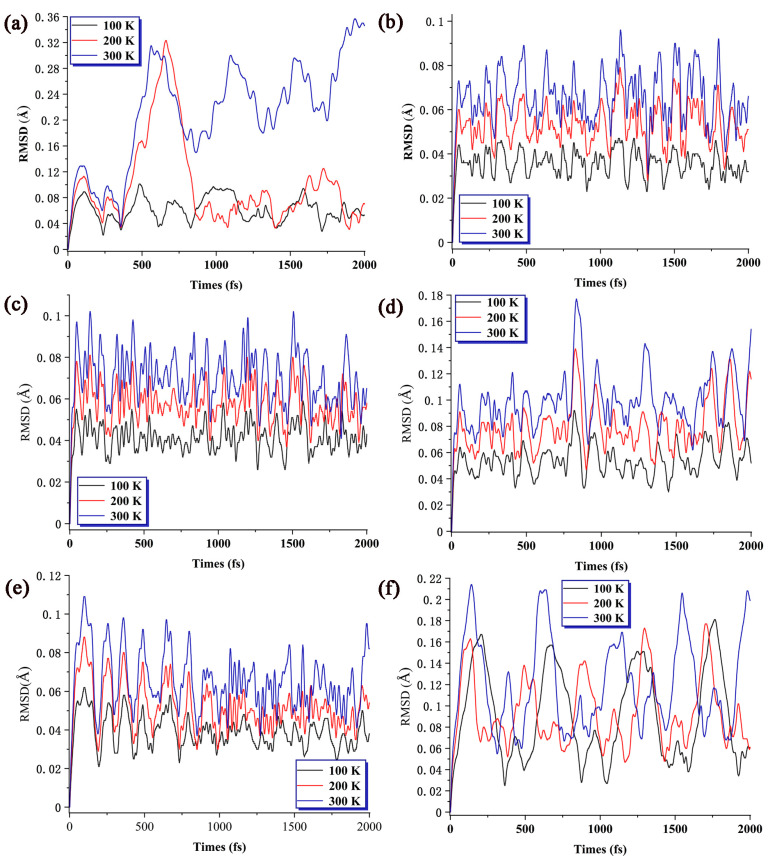
RMSD of (**a**) Nb_5_C; (**b**) Nb_5_C_2_; (**c**) Nb_5_C_6_; (**d**) Nb_5_C_7_; (**e**) Nb_6_C; and (**f**) Nb_6_C_7_ at three different temperatures. The trajectories have been aligned to the first frame prior to the RMSD calculation.

In the above discussion, through a comprehensive comparison of the results of average binding energy, dissociation energy, and second-order difference energy, we identified that Nb_5_C_2_ and Nb_5_C_6_ clusters exhibit higher stability. To analyze the reasons for the stability of these clusters, AIMD simulations, implemented in Born–Oppenheimer MD (BOMD) format, were conducted to analyze their stability. Additionally, AIMD calculations were performed on clusters positioned at the endpoints, namely Nb_5_C, Nb_5_C_7_, Nb_6_C, and Nb_6_C_7_. Each system was set at three different temperatures (100 K, 200 K, and 300 K) under vacuum conditions and maintained a certain temperature stability every 15 fs. The simulations allowed us to observe the thermal motion of the atoms and the geometric fluctuations in these systems. Figure 4 presents the root mean square deviation (RMSD) curves for the three AIMD trajectories at these temperatures. Here, RMSD is defined as the square root of the mean square difference between the position where each atom moves and its initial position. The trajectory from the beginning to the end is represented by three colors: red, white, and blue. The more overlapping of the atoms represented by these three colors, the smaller the range of atomic fluctuations, corresponding to smaller RMSD values and better thermal stability. 

As can be seen in Figure 4, by comparing the RMSD of these systems, it is evident that the order of fluctuation amplitude is Nb_5_C_2_(Nb_5_C_6_) < Nb_6_C < Nb_5_C_7_ < Nb_6_C_7_ < Nb_5_C. Neither isomerization nor dissociation of these systems was observed during 2000 fs simulations at the three temperatures, which implies that the thermal stability should never be underestimated, at least in vacuum and not too high temperature. As the temperature increases, the differences in the ability of different systems to maintain equilibrium gradually manifest. For Nb_5_C, Nb_5_C_7_, and Nb_6_C_7_, the fluctuation amplitude significantly increases with the increase in temperature, and no regular vibration period forms. Especially, the RMSD values of Nb_5_C do not show a convergence trend, indicating that it is likely to undergo dissociation. By contrast, for Nb_5_C_2_, Nb_5_C_6_, and Nb_6_C, the variation amplitude with temperature change is not significant (usually less than 0.1 Å), and it has a relatively obvious vibration period (about 250 fs) with a clear convergence trend, indicating that these structures have a certain degree of flexibility and can also maintain good stability.

**Figure 5:**
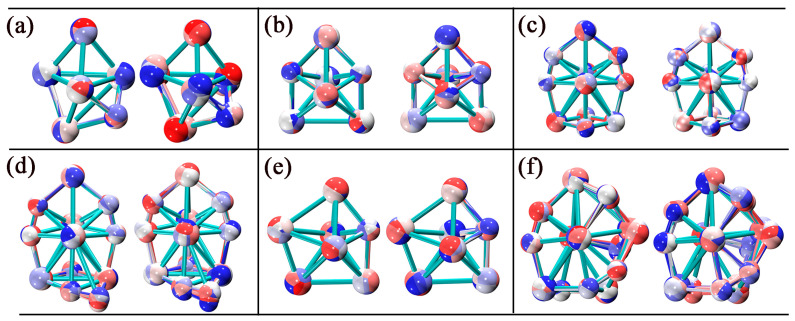
AIMD simulation trajectories of (**a**) Nb_5_C; (**b**) Nb_5_C_2_; (**c**) Nb_5_C_6_; (**d**) Nb_5_C_7_; (**e**) Nb_6_C; and (**f**) Nb_6_C_7._ The left and right sides of each small image represent the trajectories at 100 K and 300 K, respectively. The structures are extracted every 100 fs, the color corresponds to the time step and varies as red–white–blue.

The reasons for the differences in stability among the different systems can also be analyzed based on the atom colors (red–white–blue) in Figure 5. For Nb_5_C_2_ and Nb_5_C_6_, they both exhibit a certain degree of symmetry and balanced bond lengths. The horizontal movements have relatively small amplitudes, and the movements perpendicular to the paper surface are almost stationary. This results in these structures having a better ability to resist interference. On the other hand, the other candidates show a reduction or loss of symmetry, leading to a decrease in their ability to resist interference. One example is the loss of partial symmetry, as seen in Nb_6_C. Although the positioning of the doped atoms on the pentagonal ring distorts the structure of the pentagonal bipyramid, the overall structure remains largely unchanged, allowing it to maintain a certain degree of stability. The second example is the loss of symmetry through the addition of atoms, as observed in Nb_5_C_7_ and Nb_6_C_7_. The additional atoms have a greater impact on their neighboring atoms, while their impact on other atoms is relatively small, resulting in fluctuations occurring only between a portion of the atoms and minimal overall decreases in stability. The worst case is Nb_5_C, where the uneven bond lengths and fewer atoms result in weak binding forces between the atoms. Consequently, this leads to the highest volatility at high temperatures.

When comparing Figure 3, Figure 4 and Figure 5, we can observe that Nb_5_C_2_, Nb_5_C_6_, and Nb_6_C clusters, which exhibit high thermal stability, also perform well in terms of dissociation energy (E_d_) and second-order difference energy (Δ_2_E). Additionally, Nb_5_C_2_ and Nb_5_C_6_ correspond to points where the slope of the average binding energy increases rapidly, indicating that these structures are possible magic number clusters. 

### 2.5. Density of States

**Figure 6:**
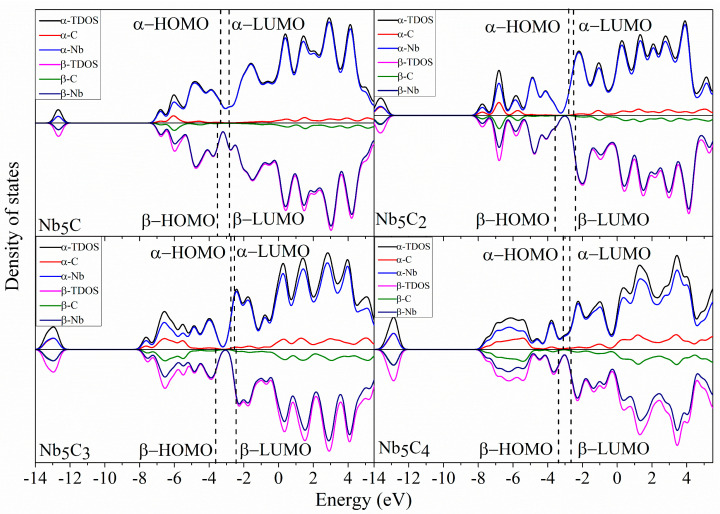
The total density of state (TDOS) and partial density of state (PDOS) of Nb_5_C_n_ (n = 1–4) with a full width at half maximum (FWHM) of 0.5 eV.

**Figure 7:**
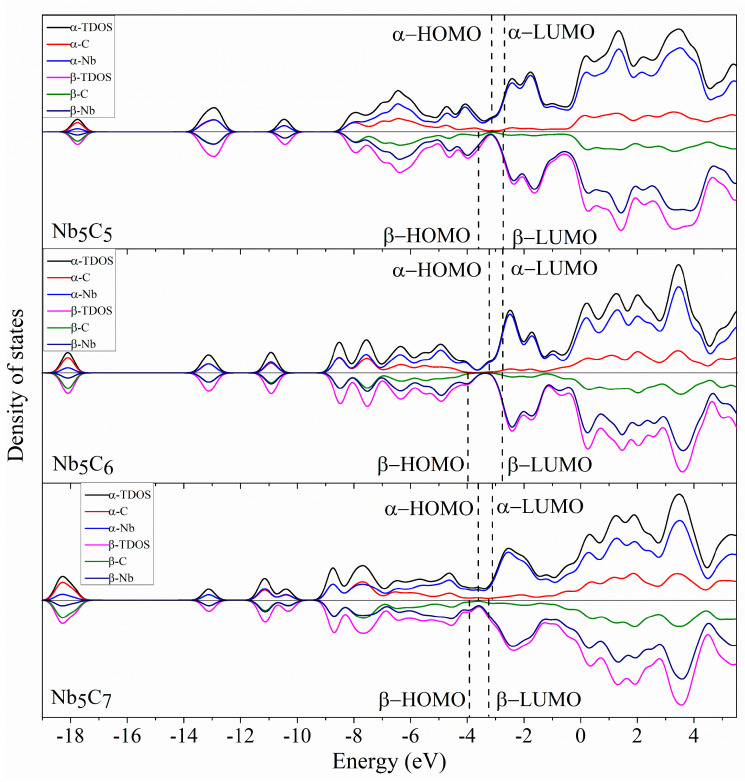
The total density of state (TDOS) and partial density of state (PDOS) of Nb_5_C_n_ (n = 5–7) with a full width at half maximum (FWHM) of 0.5 eV.

**Figure 8:**
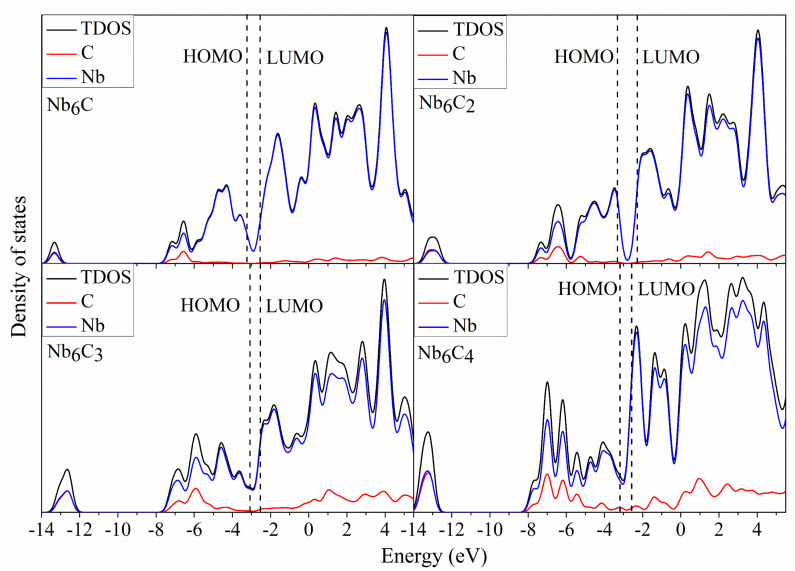
The total density of state (TDOS) and partial density of state (PDOS) of Nb_6_C_n_ (n = 1–4) with a full width at half maximum (FWHM) of 0.5 eV.

**Figure 9:**
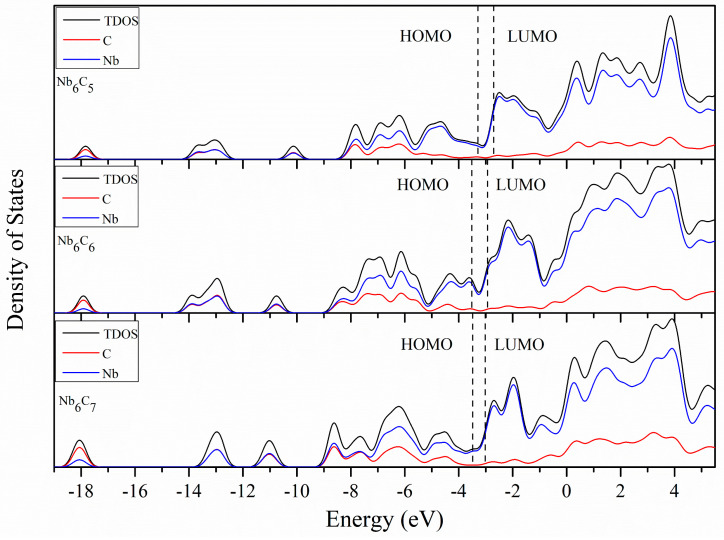
The total density of state (TDOS) and partial density of state (PDOS) of Nb_6_C_n_ (n = 5–7) with a full width at half maximum (FWHM) of 0.5 eV.

**Figure 10:**
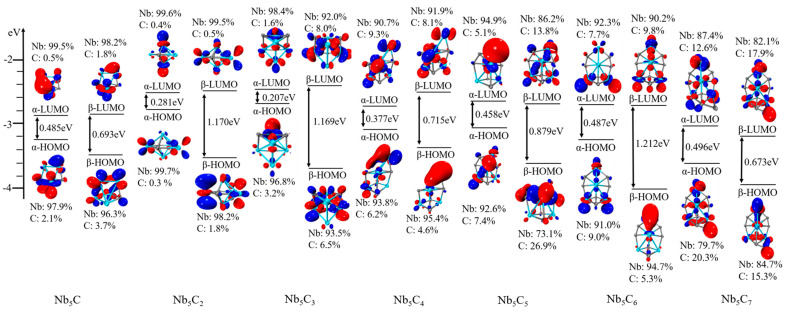
Molecular orbitals (isosurfaces = 0.05) and energy levels of Nb_5_C_n_ (n = 1–7).

**Figure 11:**
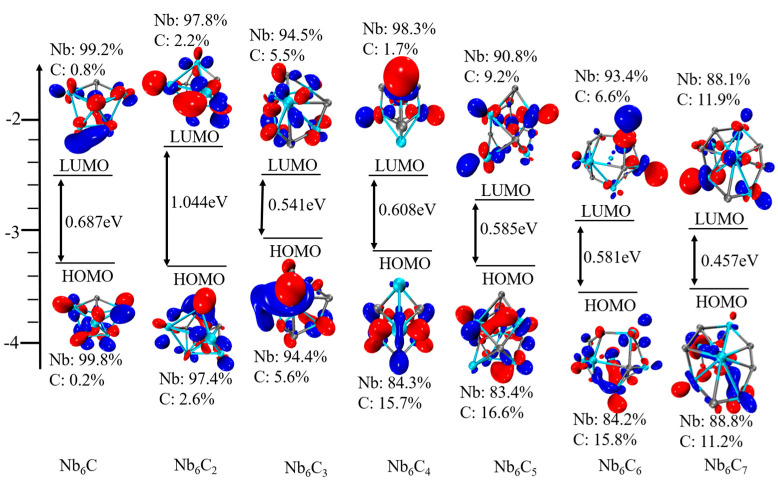
Molecular orbitals (isosurfaces = 0.05) and energy levels of Nb_6_C_n_ (n = 1–7).

To better understand the molecular orbitals of Nb_5_C_n_ and Nb_6_C_n_ (n = 1–7) clusters, the total density of states (TDOS) and the partial densities of states (PDOS) of Nb_5_/Nb_6_ and C_n_ atoms are plotted in Figure 6, Figure 7, Figure 8 and Figure 9 using the BPW91 functional. TDOS refers to the density of electronic states or the number of electronic energy levels per unit energy within a given energy range. It describes the distribution of electronic energy levels within the cluster and can be used to analyze the electronic structure of the material. The calculation of PDOS involves decomposing DOS to obtain contributions from different atomic orbitals or interatomic interactions. The calculation of Nb_5_C_n_ (n = 1–7) is based on an unrestricted open-shell treatment, and both alpha-TDOS and beta-TDOS are obtained. For Nb_6_C_n_ (n = 1–7), the calculation predicts it to be a closed shell and the spin state is singlet. From these graphs, it can be observed that for both Nb_5_C_n_ and Nb_6_C_n_ (n = 1–7) series, the PDOS profiles of the C atoms are commonly lower, compared to the PDOS curves of Nb. With the increase in the number of C atoms, the contribution of C atoms to the molecular orbitals increases slightly. That is to say, Nb atoms play an important role during the formation of molecular orbitals compared with the usually smaller contribution of C atoms. For low energy regions (the first peak), C atoms are active and contribute significantly to the molecular orbitals. However, as the energy increases, the contribution of C atoms decreases. In particular, the contribution of C atoms to the frontier molecular orbitals is very small. We provide the compositions of the frontier molecular orbitals (alpha-HOMO, alpha-LUMO, beta-HOMO, beta-LUMO) for the open-shell structures Nb_5_C_n_ (n = 1–7) and closed-shell structures Nb_6_C_n_ (n = 1–7) in Table 3 and Figure 10 and Figure 11. The results indicated that the HOMOs and LUMOs are primarily derived from the contribution of Nb atoms, and the contribution from C atoms is commonly small. For example, for α-HOMO, 79.7–99.7% is contributed by Nb atoms, while 0.3–20.3% is contributed by C atoms. For α-LUMO, 87.4–99.6% is contributed by Nb atoms, while 0.4–12.6% is contributed by C atoms. For β-HOMO, 73.1–98.2% is contributed by Nb atoms, while 1.8–26.9% is contributed by C atoms. For β-LUMO, 82.1–99.5% is contributed by Nb atoms, while 0.5–17.9% is contributed by C atoms. For the Nb_6_C_n_ (n = 1–7) cluster, 83.4–99.8% is contributed to HOMO by Nb atoms, while 0.2–16.6% is contributed to HOMO by C atoms. Additionally, 88.1–99.2% is contributed to LUMO by Nb atoms, while 0.8–11.9% is contributed to LUMO by C atoms. 

## 3. Computational Methods

The ground states of Nb_5_C_n_ and Nb_6_C_n_ (n = 1–7) clusters were determined using DFT with the generalized gradient approximation by employing GAUSSIAN programs [36]. In recent years, various algorithms and strategies have played an important role in exploring the potential energy surface (PES) of clusters [37,38,39]. We first performed the search for the global minima on the PES for Nb_5_C_n_ and Nb_6_C_n_ (n = 1–7) systems using the stochastic kicking (SK) method [40,41,42]. In recent years, our research group has effectively predicted the ground-state structures and electronic properties of binary mixed clusters utilizing the SK-DFT method [4,19,35,43]. All of the atoms are placed at the same point initially and then are “kicked” randomly with a sphere of some radius. The kick method was run approximately 500 times at the BPW91/3-21G level until no new minima appeared. Subsequently, several relevant lower-lying isomers were selected for further optimization utilizing the larger basis set. The triply-split basis set with polarization and diffuse functions, 6-311+G(2d) and SDD pseudopotential basis set, which accounts for relativistic effects, were used for C atoms and Nb atoms, respectively. The BPW91 functional was employed in these calculations [44,45], which has been successfully used in the calculations of Nb clusters [46,47,48]. Different possible spin multiplicities were also considered for each of these structural isomers to determine the preferred spin states of these complexes. For Nb_5_C_n_ (n = 1–7) clusters, spin multiplicities of 2, 4, 6, and 8 were considered during the calculation process, while for Nb_5_C_n_ (n = 1–7) clusters, spin multiplicities of 1, 3, 5, and 7 were considered. Spin-restricted DFT calculations were employed for the singlet state, while spin-unrestricted DFT calculations were employed for all other electronic states. The calculated results showed that the most stable structures of Nb_5_C_n_ and Nb_6_C_n_ (n = 1–7) prefer the lowest spin state. Vibrational frequency calculations were performed to ascertain the stability of lowest energy isomers. Hence, the structures depicted in Figure 1 represent true local minima as they exhibit positive frequencies. In addition, the low-lying energy isomers identified in Figure 1 were recalculated with higher precision using the Def2-DZVPP basis set instead of the SDD basis set, as shown in Table S2 of the Supplementary Materials. VIP is defined as the energy difference between the cation clusters at optimized neutral geometry clusters and optimized neutral clusters: VIP = E(cation clusters at optimized neutral geometry) − E(optimized neutral). AIMD simulations were performed with ORCA 5.0 code [49], and the dynamic trajectories were visualized by Visual Molecular Dynamics (VMD 1.9.3) software [50]. In the simulations, the BPW91 functional was selected, the step size was set to 1 fs, and a CSVR thermostat with a time constant of 15 fs was employed to maintain the temperature. Density of states and orbital composition analysis were performed by the Multiwfn program [51] and visualized by VMD 1.9.3 software.

## 4. Conclusions

The geometries, stabilities, and electronic properties of niobium carbide clusters, Nb_m_C_n_ (m = 5, 6; n = 1–7), have been investigated within the framework of density functional theory. The average binding energy, dissociation energy, and second-order difference energy, combined with AIMD simulations, are used to discuss the stability of Nb_m_C_n_ (m = 5, 6; n = 1–7) clusters. The results reveal that Nb_5_C_2_, Nb_5_C_6_, and Nb_6_C exhibit relatively higher thermodynamic stability among all clusters investigated in this study. In addition, the molecular orbitals of Nb_m_C_n_ (m = 5, 6; n = 1–7) are primarily contributed by niobium atoms, and niobium atoms contribute approximately 73.1–99.8% to Nb_m_C_n_ (m = 5, 6; n = 1–7) clusters. By contrast, carbon atoms make a modest contribution, ranging from 0.2% to 26.9%.

## Figures and Tables

**Table 1:** The vertical ionization potential (VIP) for Nb_5_C_n_ and Nb_6_C_n_ (n = 1–7) clusters, all energies are in eV, the values in parentheses are relative error (%).

Isomers	VIP	Expt. [28]	Isomers	VIP	Expt. [28]
5-1a	5.30 (1.0%)	5.25 ± 0.08	6-1a	5.11 (2.7%)	5.25 ± 0.07
5-2a	4.45 (3.3%)	4.6 ± 0.15	6-2a	5.20 (0.2%)	5.21 ± 0.07
5-3a	4.60 (1.5%)	4.53 ± 0.05	6-3a	4.99 (3.5%)	4.82 ± 0.06
5-4b	5.13 (0.2%)	5.12 ± 0.12	6-4a	4.94 (0.8%)	4.9 ± 0.1
5-5a	5.02 (0.6%)	5.05 ± 0.10	6-5a	5.18 (1.6%)	5.1 ± 0.1
5-6a	5.00 (0.4%)	5.02 ± 0.08	6-6a	5.28 (2.5%)	5.15 ± 0.12
5-7a	5.54 (8.6%)	5.10 ± 0.12	6-7a	5.28 (3.5%)	5.1 ± 0.12

**Table 2:** The average binding energy per atom (E_b_), dissociation energy (DE), and second-order difference energy (Δ_2_E) for Nb_5_C_n_ and Nb_6_C_n_ (n = 1–7) clusters, all energies are in eV.

Isomers	E_b_	DE	Δ_2_E	Isomers	E_b_	DE	Δ_2_E
5-1a	4.408	7.956	−0.384	6-1a	4.639	8.952	1.652
5-2a	4.970	8.340	1.151	6-2a	4.971	7.300	−0.160
5-3a	5.247	7.190	−0.333	6-3a	5.248	7.459	0.243
5-4b	5.500	7.523	−0.094	6-4a	5.444	7.216	0.111
5-5a	5.712	7.617	−0.628	6-5a	5.595	7.105	−0.279
5-6a	5.942	8.245	2.103	6-6a	5.745	7.384	−1.232
5-7a	5.959	6.142		6-7a	5.965	8.617	

**Table 3:** The compositions of the frontier molecular orbitals for open-shell structures of Nb_5_C_n_ and closed-shell structures of Nb_6_C_n_ (n = 1–7).

Isomer	Atom	α		β		Isomer	Atom	HOMO	LUMO
		HOMO	LUMO	HOMO	LUMO				
5-1a	Nb	97.9%	99.5%	96.3%	98.2%	6-1a	Nb	99.8%	99.2%
	C	2.1%	0.5%	3.7%	1.8%		C	0.2%	0.8%
5-2a	Nb	99.7%	99.6%	98.2%	99.5%	6-2a	Nb	97.4%	97.8%
	C	0.3%	0.4%	1.8%	0.5%		C	2.6%	2.2%
5-3a	Nb	96.8%	98.4%	93.5%	92.0%	6-3a	Nb	94.4%	94.5%
	C	3.2%	1.6%	6.5%	8.0%		C	5.6%	5.5%
5-4b	Nb	93.8%	90.7%	95.4%	91.9%	6-4a	Nb	84.3%	98.3%
	C	6.2%	9.3%	4.6%	8.1%		C	15.7%	1.7%
5-5a	Nb	92.6%	94.9%	73.1%	86.2%	6-5a	Nb	83.4%	90.8%
	C	7.4%	5.1%	26.9%	13.8%		C	16.6%	9.2%
5-6a	Nb	91.0%	92.3%	94.7%	90.2%	6-6a	Nb	84.2%	93.4%
	C	9.0%	7.7%	5.3%	9.8%		C	15.8%	6.6%
5-7a	Nb	79.7%	87.4%	84.7%	82.1%	6-7a	Nb	88.8%	88.1%
	C	20.3%	12.6%	15.3%	17.9%		C	11.2%	11.9%

## Data Availability

The data presented in this study are available upon request from the corresponding authors.

## Supplementary Materials

The following supporting information can be downloaded at: https://www.mdpi.com/article/10.3390/molecules29133238/s1, Table S1: Cartesian coordinates for the lowest energy structure of Nb_m_C_n_ (m = 5,6; n =1–7) at the BPW91/Nb/SDD//C/6-311+G(2d) level; Table S2: The relative energies of Nb_m_C_n_ (m = 5,6; n =1–7) at the BPW91/Nb/SDD//C/6-311+G(2d) and BPW91/Nb/Def2-TZVPP//C/6-311+G(2d) level.

## References

[1] Alonso J.A. (2000). Electronic and atomic structure, and magnetism of transition-metal clusters. Chem. Rev..

[2] Knickelvein M.B. (2001). Experimental observation of superparamagnetism in manganese clusters. Phys. Rev. Lett..

[3] Xu X.S., Yin S.Y., Moro R., De Heer W.A. (2005). Magnetic moments and adiabatic magnetization of free cobalt clusters. Phys. Rev. Lett..

[4] Fan Y.W., Kong X.Y., Zhao L.J., Wang H.Q., Li H.F., Zhan Q., Xie B., Xu H.G., Zheng W.J. (2021). A joint experimental and theoretical study on structural, electronic, and magnetic properties of MnGe_n_^−^ (n = 3–14) clusters. J. Chem. Phys..

[5] Brock L., Duncan M.A. (1996). Near-threshold photoionization to probe neutral “met-car” clusters. J. Phys. Chem..

[6] Loh S.K., Lian L., Armentrout P.B. (1989). Oxidation reactions at variably sized transition metal centers: Fe_n_^+^ and Nb_n_^+^ + O_2_ (n = 1–3). J. Chem. Phys..

[7] Hales D.A., Lian L., Armentrout P.B. (1990). Collision-induced dissociation of Nb_n_^+^ (n = 2–11): Bond energies and dissociation pathways. Int. J. Mass Spectrom. Ion Process..

[8] Cartier S.F., May B.D., Castleman A.W. (1996). The delayed ionization and atomic ion emission of binary metal metallocarbohedrenes Ti_x_M_y_C_12_ (M = Zr, Nb; 0 ≤ y ≤ 4; x + y = 8). J. Chem. Phys..

[9] Deng H.T., Guo B.C., Kerns K.P., Castleman A.W. (1994). Gas phase reactions of the met-cars Ti_8_C_12_^+^, Nb_8_C_12_^+^ and Ti_7_Nb_12_^+^ with acetone and methyl lodide. J. Phys. Chem..

[10] Yeh C.S., Byun Y.G., Afzaal S., Kan S.Z., Lee S., Freiser B.S., Hay P.J. (1995). Experimental and theoretical studies on Nb_4_C_4_^0/+^: Reactivity and structure of the smallest cubic niobium-carbon cluster. J. Am. Chem. Soc..

[11] Byun Y.G., Lee S.A., Kan S.Z., Freiser B.S. (1996). Reactivities of metallocarbohedrenes: Nb_8_C_12_^+^. J. Phys. Chem..

[12] Yang D.S., Zgierski M.Z., Berces A., Hackett P.A., Roy P.N., Martinez A., Carrington T., Salahub D.R., Fournier R., Pang T. (1996). Vibrational and geometric structures of Nb_3_C_2_ and Nb_3_C_2_^+^ from pulsed field ionization-zero electron kinetic energy photoelectron spectra and density functional calculations. J. Chem. Phys..

[13] Li S., Wu H., Wang L. (1997). Probing the electronic structure of metallocarbohedrenes: M_8_C_12_ (M = Ti, V, Cr, Zr, Nb). J. Am. Chem. Soc..

[14] Knappenberger K.L., Clayborne P.A., Reveles J.U., Sobhy M.A., Jones C.E., Gupta U.U., Khanna S.N., Iordanov I., Sofo J., Castleman A.W. (2007). Anion photoelectron spectroscopy and density functional investigation of diniobium-carbon clusters. ACS Nano.

[15] Geusic M.E., Morse M.D., Smalley R.E. (1985). Hydrogen chemisorption on transition metal clusters. J. Chem. Phys..

[16] Whetten R.L., Zakin M.R., Cox D.M., Trevor D.J., Kaldor A. (1986). Electron binding and chemical inertness of specific Nb_x_ clusters. J. Chem. Phys..

[17] Eduok U. (2020). Niobia Nanofiber-Reinforced Protective Niobium Oxide/Acrylate Nanocomposite Coatings. ACS Omega.

[18] Korzyński M.D., Xie L.S., Dincă M. (2020). Structural Characterization of a High-Nuclearity Niobium(V) Carboxylate Cluster Based on Pivalic Acid. Helv. Chim. Acta.

[19] Wang H.Q., Li H.F. (2012). Probing the structural and electronic properties of al-doped small niobium clusters. Chem. Phys. Lett..

[20] Grönbeck H., Rosén A., Andreoni W. (1998). Structural electronic, and vibrational properties of neutral and charged Nb_n_ (n = 8, 9, 10) clusters. Phys. Rev. A.

[21] Pansinia F.N.N., Camposb M.D., Netoa A.C., Sergiob C.S. (2020). Theoretical study of the electronic structure and electrical properties of Al-doped niobium clusters. Chem. Phys..

[22] Prudnikava A., Tamashevich Y., Babenkov S., Makarova A., Smirnov D., Aristov V., Molodtsova O., Kugeler O., Viefhaus J., Foster B. (2022). Systematic study of niobium thermal treatments for superconducting radio frequency cavities employing x-ray photoelectron spectroscopy. Supercond. Sci. Technol..

[23] Oliveira L., Pereira M., Heitman A.P., Filho J., Olivira C., Ziolek M. (2023). Niobium: The focus on catalytic application in the conversion of biomass and biomass derivatives. Molecules.

[24] Luo P., Zhao Y.H. (2023). Niobium nitride preparation for superconducting single-photon detectors. Molecules.

[25] Wei S., Guo B.C., Deng H.T., Kerns K., Purnell J., Buzza S.A., Castleman A.W. (1994). Formation of met-cars and face-centered cubic structures: Thermodynamically or kinetically controlled?. J. Am. Chem. Soc..

[26] Pilgrim J.S., Brock L.R., Duncan M.A. (1995). Photodissociation of niobium-carbon clusters and nanocrystals. J. Phys. Chem..

[27] Dryza V., Addicoat M.A., Gascooke J.R., Buntine M.A., Metha G.F. (2008). Threshold photoionization and density functional theory studies of the niobium carbide clusters Nb_3_C_n_ (n = 1–4) and Nb_4_C_n_ (n = 1–6). J. Phys. Chem. A.

[28] Fukushima N., Miyajima K., Mafune F. (2009). Ionization energies of niobium carbide clusters Nb_n_C_m_ (n = 3–10, m = 0–7). J. Phys. Chem. A.

[29] Gusev A.I. (2020). Anisotropy of microstructure and elastic properties of niobium carbide nanopowders. Solid State Sci..

[30] Chebanenko M.I., Danilovich D.P., Lobinsky A.A., Popkov V.I., Rempel A.A., Valeeva A.A. (2021). Novel high stable electrocatalyst based on non-stoichiometric nanocrystalline niobium carbide toward effective hydrogen evolution. Int. J. Hydrogen Energy.

[31] Zhai H.J., Liu S.R., Wang L.S. (2001). Photoelectron spectroscopy of mono-niobium carbide clusters NbC_n_^−^ (n= 2–7): Evidence for a cyclic to linear structural transition. J. Chem. Phys..

[32] Il’in E.G., Parshakov A.S., Teterin Y.A., Maslakov K.I., Teterin A.Y. (2020). Surface morphology and composition of a NbC/C composite studied by scanning electron microscopy and X-ray photoelectron spectroscopy. Inorg. Mater..

[33] Hamrick Y.M., Weltner W. (1991). Quenching of angular momentum in the ground states of VC, NbC, VSi, and NbSi molecules. J. Chem. Phys..

[34] Valeeva A.A., Gusev A.I. (2021). Effect of nonstoichiometry on elastic properties of niobium carbide NbC_y_. Int. J. Refract. Met. H..

[35] Li H.F., Wang H.Q., Zhang J.M., Qin L.X., Zheng H., Zhang Y.H. (2024). Investigation of structures, stabilities, and electronic and magnetic properties of niobium carbon clusters Nb_7_C_n_ (n = 1–7). Molecules.

[36] Frisch M.J., Trucks G.W., Schlegel H.B., Scuseria G.E., Robb M.A., Cheeseman J.R., Scalmani G., Barone V., Mennucci B., Petersson G.A. (2010). Gaussian 09, Revision C.01.

[37] Diego I., Alejandro V.S., Luis L.P., Williams G.A., Maria L.C., Osvaldo Y., William T. (2023). Revisiting the potential-energy surface of C_n_Be_3n+2_H_2n+2_^2+^ (n = 2–4) clusters: Are planar pentacoordinate carbon structures the global minima?. Phys. Chem. Chem. Phys..

[38] Venkatesan S.T., Aland S., Diego I., Pothiappan V., Krishnan T., Saikat R., Anakuthil A., William T. (2022). Why an integrated approach between search algorithms and chemical intuition is necessary?. Phys. Chem. Chem. Phys..

[39] Osvaldo Y., Diego I., Brandon U.A., Alejandro V.E., Ricardo P.R., Mauricio T.S., Jorge G., Jorge B., Gabriel M., William T. (2020). Evaluation of restricted probabilistic cellular automata on the exploration of the potential energy surface of Be_6_B_11_^−^. Theor. Chem. Acc..

[40] Roy D., Corminboeuf C., Wannere C.S., King R.B., Schleyer P.V.R. (2006). Planar tetracoordinate carbon atoms centered in bare four-membered rings of late transition metals. Inorg. Chem..

[41] Bera P.P., Sattelmeyer K.W., Saunders M., Schaefer H.F., Schleyer P.R. (2006). Mindless chemistry. J. Phys. Chem. A.

[42] Saunders M. (2004). Stochastic search for isomers on a quantum mechanical surface. J. Comput. Chem..

[43] Li H.F., Wang H.Q. (2018). Stabilization of golden cages by encapsulation of a single transition metal atom. R. Soc. Open Sci..

[44] Becke A.D. (1988). Density-functional exchange-energy approximation with correct asymptotic behavior. Phys. Rev. A.

[45] Perdew J.P., Wang Y. (1992). Accurate and simple analytic representation of the electron-gas correlation energy. Phys. Rev. B.

[46] Huang B.B., Zhang H.Y., Gan W., Yang M.Z., Luo Z.X., Yao J.N. (2023). Nb_12_^+^—Niobespherene: A full-metal hollow-cage cluster with superatomic stability and resistance to CO attack. Natl. Sci. Rev..

[47] Pham V.N., Vu T.N., Truong B.T., Minh T.N. (2011). Electronic Structures, Vibrational and Thermochemical Properties of Neutral and Charged Niobium Clusters Nb_n_, n = 7–12. J. Phys. Chem. A.

[48] Pham V.N., Vu T.N., Minh T.N. (2010). A New Look at the Structure and Vibrational Spectra of Small Niobium Clusters and Their Ions. J. Phys. Chem. C.

[49] Neese F. (2022). Software update: The ORCA program system-Version 5.0. Wiley Isnterdiscip. Rev. Comput. Mol. Sci..

[50] Humphrey W., Dalke A., Schulten K. (1996). VMD: Visual molecular dynamics. J. Mol. Graph..

[51] Lu T., Chen F.W. (2012). Multiwfn: A multifunctional wavefunction analyzer. J. Comput. Chem..

