# Supplementary material for: Exploring the Structural and Electronic Properties of Niobium Carbide Clusters: A Density Functional Theory Study
Source: Molecules. 2024 Jul 8;29(13):3238. doi: 10.3390/molecules29133238 (PMC11243446; doi:10.3390/molecules29133238)
Supplement: Supplementary file 1 [file molecules-29-03238-s001.zip › molecules-3069370-supplementary.pdf]

Supplementary Materials

Exploring the Structural and Electronic Properties of Niobium Carbide Clusters: A Density Functional Theory Study

Hui-Fang Li [1], Huai-Qian Wang [1,2,*] and Yu-Kun Zhang [1]

[1] College of Engineering, Huaqiao University, Quanzhou 362021, China
[2] College of Information Science and Engineering, Huaqiao University, Xiamen 361021, China
[*] Correspondence: hqwang@hqu.edu.cn

Table of Contents

Table S1. Cartesian coordinates for the lowest energy structure of Nb_mC_n (m = 5,6; n =1-7) at the BPW91/Nb/SDD//C/6-311+G(2d) level.

Table S2. The relative energies of Nb_mC_n (m = 5,6; n =1-7) at the BPW91/Nb/SDD//C/6-311+G(2d) and BPW91/Nb/ Def2-TZVPP //C/6-311+G(2d) level. The relative energies are in eV.

Table S1. Cartesian coordinates for the lowest energy structure of Nb_mC_n (m = 5,6; n =1-7) at the BPW91/Nb/SDD//C/6-311+G(2d) level.

Nb_5C

Atom	X	Y	Z
Nb	0.083742	-0.985677	-1.243375
Nb	-1.895594	0.257887	-0.400048
Nb	2.003850	0.024844	0.045283
Nb	-0.545576	-0.661454	1.503541
Nb	0.228781	1.607052	0.008531
C	0.852773	-1.658124	0.588131

Nb_5C_2

Atom	X	Y	Z
Nb	-0.114280	-1.562581	-0.000465
Nb	-2.215250	0.000250	0.000008
Nb	1.327445	0.000296	-1.419672
Nb	-0.113609	1.562624	0.000536
Nb	1.327521	-0.000638	1.419595
C	-0.723790	0.000750	-1.401197
C	-0.723696	-0.000409	1.401182

Nb_5C_3

Atom	X	Y	Z
Nb	-1.842072	-0.143463	-0.280330
Nb	0.092125	-1.808704	-0.656585
Nb	1.958842	0.147623	-0.173522
Nb	-0.207509	1.800901	-0.658147
C	0.899944	1.450731	0.995193
C	0.100252	0.009171	-1.487267
Nb	-0.310975	-0.020974	1.692966
C	1.115328	-1.291676	1.008797

Nb_5C_4-a

Atom	X	Y	Z
Nb	-1.541128	-1.262655	0.000000
Nb	0.295021	-0.629508	1.665713
Nb	1.701891	1.197783	0.000000
Nb	0.295021	-0.629508	-1.665713
C	0.295021	1.455668	-1.389259
C	0.624230	-1.914546	0.000000

Nb	-1.168456	1.303579	0.000000
C	0.295021	1.455668	1.389259
C	1.639667	-0.858011	0.000000

Nb_5C_4-b

Atom	X	Y	Z
Nb	0.380468	-0.466074	-1.328851
C	-0.532004	-1.748733	0.000042
C	1.213596	1.203944	-0.000015
Nb	-0.796934	1.699565	-0.000036
Nb	-2.269698	-0.766930	0.000006
Nb	0.380451	-0.466009	1.328882
Nb	2.669842	-0.060197	0.000000
C	-1.584890	0.476153	-1.386095
C	-1.584912	0.476212	1.386061

Nb_5C_5

Atom	X	Y	Z
Nb	-2.485859	0.200155	-0.010571
Nb	-0.399328	-0.227455	1.371104
Nb	1.868724	-1.140235	0.018722
Nb	-0.369210	-0.268045	-1.367669
C	1.722088	0.299695	-1.371926
C	-1.422218	-1.696675	0.006448
Nb	1.229907	1.682579	-0.013475
C	1.695504	0.323615	1.380025
C	-0.114876	-2.077657	0.022829
C	-0.816089	1.463197	-0.024472

Nb_5C_6

Atom	X	Y	Z
Nb	-1.788714	1.433784	-0.000169
Nb	0.396870	-0.000182	1.258550
C	-1.808301	0.000089	-1.381920
C	0.210868	-1.911894	0.000377
C	1.540920	1.595793	0.000950
Nb	0.397984	0.000180	-1.258143
C	-1.809148	-0.000081	1.381286
Nb	2.799264	-0.000012	-0.000064

C	0.210903	1.911930	0.000479
Nb	-1.788742	-1.433775	-0.000424
C	1.540902	-1.595807	0.000544

Nb_5C_7

Atom	X	Y	Z
Nb	-1.753016	-1.316099	-0.362253
Nb	0.530227	0.243474	-1.297443
C	-2.051992	-0.036218	1.450037
C	0.345515	1.884842	0.320841
C	1.557937	-1.589012	-0.270345
Nb	0.336248	-0.180037	1.199032
C	-1.593013	0.402323	-1.559200
Nb	2.868989	-0.108241	0.152164
C	0.225738	-1.815103	-0.441132
Nb	-1.651497	1.501663	0.096572
C	1.655511	1.502384	0.318531
C	-2.401186	-1.311075	1.629438

Nb_6C

Atom	X	Y	Z
Nb	0.000000	1.909436	1.067422
Nb	0.000000	-1.909436	1.067422
Nb	-1.597444	-0.041842	0.332012
Nb	-0.345868	1.166757	-1.530438
Nb	0.345868	-1.166757	-1.530438
Nb	1.597444	0.041842	0.332012
C	0.000000	0.000000	1.790379

Nb_6C_2

Atom	X	Y	Z
Nb	1.219450	1.511194	-0.708949
Nb	-0.546659	-1.077256	1.325790
Nb	-1.887742	-0.560144	-0.789000
C	-0.522034	0.862660	-1.311238
Nb	-1.219518	1.510997	0.709186
Nb	0.546716	-1.076997	-1.325970
Nb	1.887758	-0.560242	0.788925
C	0.522007	0.862405	1.311361

Nb_6C_3

Atom	X	Y	Z
Nb	-0.075807	-1.867114	0.727628

Nb	1.928741	-0.202049	-0.614314
Nb	-0.331345	-0.812474	-1.625418
Nb	-2.016336	0.112668	0.090264
C	-1.171966	-0.451310	1.762043
C	1.604360	1.672764	-0.005505
Nb	0.444547	0.863913	1.660752
Nb	-0.242318	1.853326	-0.677561
C	1.566481	-0.867955	1.240905

Nb_6C_4

Atom	X	Y	Z
Nb	-1.592727	0.000839	-0.705344
Nb	0.000000	1.592740	0.705342
C	1.399703	0.000085	1.417968
C	0.000908	1.399696	-1.417928
Nb	0.000000	-1.592740	0.705342
Nb	1.592727	-0.000839	-0.705344
Nb	0.000000	0.000000	2.849816
C	-1.399703	-0.000085	1.417968
Nb	0.000000	0.000000	-2.849823
C	-0.000908	-1.399696	-1.417928

Nb_6C_5

Atom	X	Y	Z
Nb	-0.621134	-1.007257	1.389243
Nb	0.793433	-1.287589	-1.196991
Nb	2.073820	-0.467471	0.859982
Nb	1.491087	1.462330	-0.686034
Nb	-0.930346	1.716746	0.369154
Nb	-1.953382	-0.465485	-0.893609
C	-0.760488	-2.158316	-0.291013
C	0.639369	0.906141	1.291100
C	-2.354721	0.224096	1.216669
C	-0.218547	0.585344	-1.416812
C	-3.137712	0.775697	0.281461

Nb_6C_6

Atom	X	Y	Z
Nb	2.528549	-0.585262	-0.369746
Nb	-2.90855	-0.070452	-0.61445
Nb	-0.207464	-0.053749	-1.112673
Nb	0.232980	-1.970618	0.715934
Nb	1.327997	1.936874	0.288872

Nb	-1.006814	0.502240	1.302264
C	1.996754	1.073548	-1.317546
C	-1.656037	-1.323761	0.315224
C	-1.851243	1.622663	-0.424525
C	-0.646681	2.177075	-0.097280
C	1.193941	-1.859792	-1.046427
C	1.190828	-0.043127	1.134178

Nb_6C_7

Atom	X	Y	Z
Nb	1.853069	1.344558	-0.322296
Nb	-0.984942	1.811623	0.450005
Nb	-0.448418	-0.624488	1.364286
Nb	2.052800	-1.472323	0.254167
Nb	-0.050305	-0.206764	-1.429249
Nb	-2.630122	-0.701005	-0.49777
C	1.623180	0.122514	1.532972
C	2.243283	-0.361612	-1.332559
C	0.115102	1.861602	-1.258086
C	-1.096741	-1.972728	-0.327352
C	-2.381229	0.373666	1.065949
C	0.703627	1.198571	1.592611
C	0.213552	-2.257946	-0.037668

Table S2. The spin multiplicity M and the relative energies ΔE of Nb_mC_n (m = 5,6; n =1-7) at the BPW91/Nb/SDD//C/6-311+G(2d) and BPW91/Nb/ Def2-TZVPP //C/6-311+G(2d) level. The relative energies are in eV.

Isomers	M	ΔE		Isomers	M	ΔE	
		SDD	Def2-TZVPP			SDD	Def2-TZVPP
5-1a	2	0.00	0.00	6-1a	1	0.00	0.00
5-1b	2	0.53	0.54	6-1b	1	0.66	0.61
5-1c	2	0.82	0.82	6-1c	1	0.99	1.01
5-2a	2	0.00	0.00	6-2a	1	0.00	0.00
5-2b	2	0.69	0.72	6-2b	1	0.67	0.64
5-2c	2	0.93	0.94	6-2c	1	0.85	0.80
5-3a	2	0.00	0.00	6-3a	1	0.00	0.00
5-3b	2	0.30	0.31	6-3b	1	0.50	0.49
5-3c	2	0.53	0.50	6-3c	1	0.64	0.61
5-4a	2	0.00	0.01	6-4a	1	0.00	0.00
5-4b	2	0.02	0.00	6-4b	1	0.75	0.88
5-4c	2	0.15	0.12	6-4c	1	0.95	0.98
5-5a	2	0.00	0.00	6-5a	1	0.00	0.00
5-5b	2	0.45	0.47	6-5b	1	0.24	0.24
5-5c	2	1.03	1.02	6-5c	1	0.43	0.46
5-6a	2	0.00	0.00	6-6a	1	0.00	0.00
5-6b	2	1.27	1.25	6-6b	1	0.73	0.82
5-6c	2	1.75	1.76	6-6c	1	0.84	0.89
5-7a	2	0.00	0.00	6-7a	1	0.00	0.00
5-7b	2	0.07	0.06	6-7b	1	1.75	1.70
5-7c	4	0.25	0.26	6-7c	1	2.31	2.31
